# EPIC-NET: EEG-based epilepsy classification and brain localization using Optuna wave-gated recurrent unit network

**DOI:** 10.3389/fncom.2025.1725924

**Published:** 2026-02-03

**Authors:** R. Manjupriya, A. Anny Leema

**Affiliations:** 1 School of Computer Science Engineering and Information Systems, Vellore Institute of Technology, Vellore, Tamil Nadu, India; 2 School of Computer Science and Engineering, Vellore Institute of Technology, Vellore, Tamil Nadu, India

**Keywords:** electroencephalography signals, epilepsy detection, fully connected layer, Optuna wave-gated recurrent unit, ResGoogleNet

## Abstract

**Introduction:**

Epilepsy is a chronic neurological disorder characterized by abnormal brain activity, often diagnosed through visual analysis of electroencephalography (EEG) signals. However, the existing works focused only on general epilepsy and failed to focus on location-based wave detection.

**Methods:**

In this work, a novel deep learning-based EPIC-NET is proposed for epilepsy classification and brain localization using EEG signal. The EEG signals are fed into ResGoogleNet to extract both temporal and spatial features such as frequency variations, waveform morphology, and amplitude changes for epilepsy detection and localization of the affected brain regions. Stochastic Variance Reduced Gradient Langevin Dynamics based Honey Badger (SVGL-HBO) algorithm is utilized for feature selection effectively reducing dimensionality and retaining the most relevant features for detection. Based on the selected features, a fully connected layer classifies the normal and epilepsy. The Seizure Activity Index of epilepsy is classified into Low, Medium, and High using a Bell Elliptic Fuzzy Logic System (BE-FLS) guided by predefined fuzzy rules. The Optuna Wave-Gated Recurrent Unit (OW-GRU) combines GRU with wavelet processing to extract both temporal and frequency-domain features from EEG signals. Optuna is used for automatic hyperparameter tuning, which improves GRU performance, reduces overfitting, and enables accurate localization of epilepsy within specific brain lobes.

**Results:**

The proposed EPIC-NET achieves the classification accuracy (CA) of 98.80% and Matthews Correlation Coefficient (MCC) of 97.43%.

**Discussion:**

The EPIC-NET model improves the overall accuracy by 5.92, 10.02, and 0.59% better than RNN, SVM and CNN, respectively.

## 1 Introduction

Epilepsy is a neurological condition characterized by frequent seizures caused by an excess of electrical activity in brain cells. A survey conducted by the World Health Organization (WHO) revealed that more than 50 million people suffer from epilepsy (Najafi et al., 2022; Kala et al., 2025). There are various types of epilepsy, and some individuals can identify a specific cause and others may remain unaware of it. The symptoms of seizures can include loss of consciousness, muscle stiffness, and temporary confusion. The frequency and intensity of seizures can vary significantly among individuals, affecting daily life and overall health (Ein Shoka et al., 2023; Supriya et al., 2021). Therefore, early detection and treatment are essential. Most people with epilepsy can manage their seizures through medications, while some may require surgery and a few needs lifelong treatment (Islam et al., 2023; George et al., 2020).

EEG analysis is commonly used to diagnose epilepsy by recognizing unusual brain activity rhythms (Ahilan et al., 2023). This helps to identify the seizure condition and assess the types and patterns of seizures that occur in the brain, which is crucial for effective management and treatment (Safdar and Cheng, 2024; Saminu et al., 2021). EEG is a non-invasive and frequently used method for identifying abnormal brain activity, especially for the early identification of seizures (Raghuram et al., 2024). This non-invasive method provides important insights into epilepsy and other neurological disorders (Qin et al., 2020; Tsiouris et al., 2018). Thus, the algorithms for Deep Learning (DL) (Bindhu et al., 2023) and Machine Learning (ML) (Sundarasekar and Appathurai, 2022) are utilized to detect epileptic episodes, with the analysis of EEG signal features significantly influencing detection outcomes (Nkengfack et al., 2020). Most of the existing research methodologies used ML approaches like Support Vector Machines (SVM) and Random Forest (RF) for detecting seizures (Brari and Belghith, 2021). The reliability and efficiency of RF and SVM make them suitable for real time monitoring, enhancing epilepsy management and patient outcomes (Miltiadous et al., 2022).

Deep learning models, such as Long Short-Term Memory (LSTM) networks, capture temporal relationships in EEG signals, allowing for automated feature learning and effective seizure identification. It also improved the prediction accuracy and was robust against noise in EEG recordings (Tawhid et al., 2022; Mir et al., 2023). Most existing research has focused primarily on epilepsy detection, but has largely overlooked the localization of epileptic seizures within specific brain lobes. Therefore, this research methodology proposes an enhanced detection of epilepsy based on waveform localization. The primary contribution of this work is the development of a unified multi-task learning framework, EPIC-NET, that addresses seizure detection, Seizure Activity Index estimate, and brain-lobe localization from raw EEG data inside a single architecture. Unlike previous research, which treated both tasks independently or focused exclusively on detection, EPIC-NET incorporates complementary learning components customized to each objective while sharing a common feature representation. A modified ResGoogleNet backbone is used for multi-scale feature extraction, SVGL-HBO is utilized to refine discriminative characteristics for detection, a neuro-symbolic Bell Elliptic Fuzzy Logic System allows for interpretable Seizure Activity Index classification, and an Optuna-optimized Wave-GRU facilitates temporal modeling for lobe-level localization. EPIC-NET’s uniqueness is not in the individual components themselves, but in their fundamental architectural integration into a coherent framework that allows for simultaneous, interpretable, and patient-independent epilepsy investigation. The main contributions of the research are as follows:

- The Dual Tree Complex Wavelet Transform (DT-CWT) is employed for removing artifacts and noise from EEG signals. By preserving essential neurological patterns for ensuring a clean and reliable signal for accurate analysis.
- Signal augmentation techniques such as time stretching, pitch shifting, and noise addition are applied to EEG signals to balance class distribution and enhance the model generalization across different epileptic patterns.
- The augmented spectrograms are fed into ResGoogleNet for deep feature extraction, where ResNet residual connections preserve essential low-level features, and GoogleNet inception modules capture multi-scale patterns.
- SVGL-HBO algorithm enhances classification performance by optimizing feature selection through a balance of exploration and exploitation and a FC layer classifies as normal or epileptic.
- The BE-FLS determines the Seizure Activity Index of epilepsy and based on these stages, the OW-GRU classifies the epileptic signals into affected brain regions like frontal, temporal, occipital and parietal by integrating a wavelet-enhanced GRU with Optuna-based hyperparameter tuning.

The structure of the paper is organized as follows, Section 2 presents a comprehensive review of existing research and methodologies related to detecting and localizing epilepsy, the proposed EPIC-NET was explained in section 3, the performance outcomes and their comparison analysis were provided in section 4 and section 5 comprises with conclusion and future work.

## 2 Literature survey

Researchers have developed various approaches for classifying epileptic activity from EEG signals. Advanced DL and ML techniques have been explored to improve the reliability of detecting and localizing epilepsy. Some of these methods are reviewed in this section. In 2022, Emara et al. (2022) proposed an effective technique for epileptic seizure detection by combining Scale Invariant Feature Transform (SIFT) and Fast Fourier Transform (FFT) for robust EEG feature extraction with accuracy of 90.90%, highlighting its potential as a reliable tool for automated seizure detection. In 2021, Lu et al. (2021) suggested a non-linear dynamic framework for detecting and classifying epileptic EEG signals utilizing SVM. The model achieved a notable accuracy rate of 89.8%, indicating its effectiveness on smaller datasets. However, the framework faced challenges with overfitting, limiting its generalization capability. Additionally, it struggled to handle large-scale EEG datasets efficiently.

In 2021, Yazid et al. (2021) suggested a ML model for feature extraction using the Local Binary Pattern Mean Absolute Deviation (LBPMAD) and Local Binary Pattern Transition Histogram (LBPTH). However, it was noise- sensitive to noise, leading to potential information loss. It achieved a high accuracy of 99.1% with compact feature vector size. Despite its high accuracy, the method lacked robustness when applied to large-scale, diverse EEG datasets. In 2022, Sunaryono et al. (2022) suggested an automatic epilepsy detection method using Discrete Wavelet Transform (DWT) for feature extraction, and the Gradient Boosting Machine (GBM) for EEG signals classification. The result indicated that the GBM detected the epilepsy with higher accuracy. But it still did not focus on removing the noise from the signals.

In 2022, Rashed-Al-Mahfuz et al. (2021) suggested a Fine-Tuned Visual Geometry Group (FTVGG16) technique for identifying seizures and distinctive frequencies. It achieved the highest accuracy when compared with the existing methods but had overfitting issues and high computational complexity. Moreover, its performance heavily depended on large training datasets to maintain accuracy. In 2021, Mandhouj et al. (2021) suggested CNN based technique for epilepsy diagnosis based on the spectrogram. Important elements from EEG data were extracted using the Short-Time Fourier Transform (STFT). CNN achieved better performance with higher accuracy with other models. But it was still slower because of the max pool operations. Additionally, the model’s computational demand limited its suitability for real-time clinical applications.

In 2024, Wang et al. (2024) suggested a LSTM networks for the early detection of epilepsy. The model was designed to extract both temporal and spatial features from EEG signals, which are critical for accurately identifying seizure patterns. By leveraging the sequential learning capability of LSTM, the framework aimed to detect epileptic activity at an early stage. The approach demonstrated high accuracy and robustness, making it suitable for real-time clinical applications. In 2020, Urbina Fredes et al. (2024) suggested an SVM based model for wavelet-based analysis of the EEG signals to automatically predict epileptic seizures. The presented model showed satisfactory precision and reliability in detecting seizures and achieved an accuracy rate of 92.82%. However, SVM could not process large datasets effectively. Additionally, the model’s performance was limited by its inability to adapt to varying patient-specific EEG patterns.

In 2024, Shah et al. (2024) suggested a DL framework consist of discrete wavelet transform and Random Neural Networks (RNN) for classifying epileptic seizures using of the EEG signals. RNN achieved the highest classification accuracies with other models. But, the information leakage still occurred while combining the EEG data from different patients, highlighting the need for improved data separation and patient-independent validation strategies. In 2021, Malekzadeh et al. (2021) proposed a DL approach for the automatic detection of epileptic seizures using a computer-aided diagnosis system. The method combined CNN and RNN to effectively extract spatial and temporal features from EEG signals. This hybrid architecture enhanced the AC and robustness of seizure detection demonstrating superior performance compared to traditional single-model approaches. In 2025, Sikarwar et al. (2025) introduced an entropy-driven deep learning system for epilepsy diagnosis utilizing EEG recordings, combining entropy-based characteristics with deep neural networks to improve seizure discriminate accuracy and robustness. In 2025, Raj et al. (2025) introduced an unsupervised learning framework for identifying autism spectrum disorder (ASD) subtypes with morphological characteristics collected from structural MRI (sMRI), proving the efficacy of data-driven feature clustering for neurodevelopmental disease research. In 2024, Jain et al. (2024) proposed age- and Seizure Activity Index-related deep learning techniques for autism spectrum disorder classification based on functional connectivity measures, indicating that categorized modeling improves classification accuracy by reflecting heterogeneity across age groups and Seizure Activity Index levels. In 2023, Jain et al. (2023) proposed an age-specific deep learning-based diagnostic paradigm for ASD classification, emphasizing the necessity of age-aware modeling in capturing developmental differences in brain patterns and improving classification performance. In 2023, Gupta et al. (2023) developed a system for ASD detection that uses morphological connectivity characteristics collected from structural MRI and categorized using XGBoost, resulting in better diagnostic performance through feature-driven machine learning. From the literature review, various existing models were explored with different DL architectures for epileptic seizure detection and brain lobe localization.

Existing research on EEG-based epilepsy analysis has primarily focused on seizure identification as a standalone task, using either handmade feature-based classical classifiers or deep learning models tuned for binary classification. While recent research has investigated into patient-independent evaluation and, in certain circumstances, attention-enhanced recurrent or transformer-based designs to increase temporal modeling and interpretability. Clinically relevant expansions, such as seizure activity classification or spatial localization, have received little attention and are often addressed via separate *post-hoc* studies or simplified channel-level heuristics. In contrast, the present research enriches the existing literature by defining seizure detection, interpretable seizure activity indexing, and brain-lobe localization as simultaneous learnable objectives within a single multi-task and neuro-symbolic learning paradigm. EPIC-NET advances beyond single-task detection models by combining deep spatiotemporal feature recognition with symbolic reasoning within a patient-independent evaluation process. This paper proposes an innovative method to address seizure detection and localization by merging a BE-FLS with an OW-GRU.

## 3 Proposed EPIC-NET methodology

**Figure 1:**
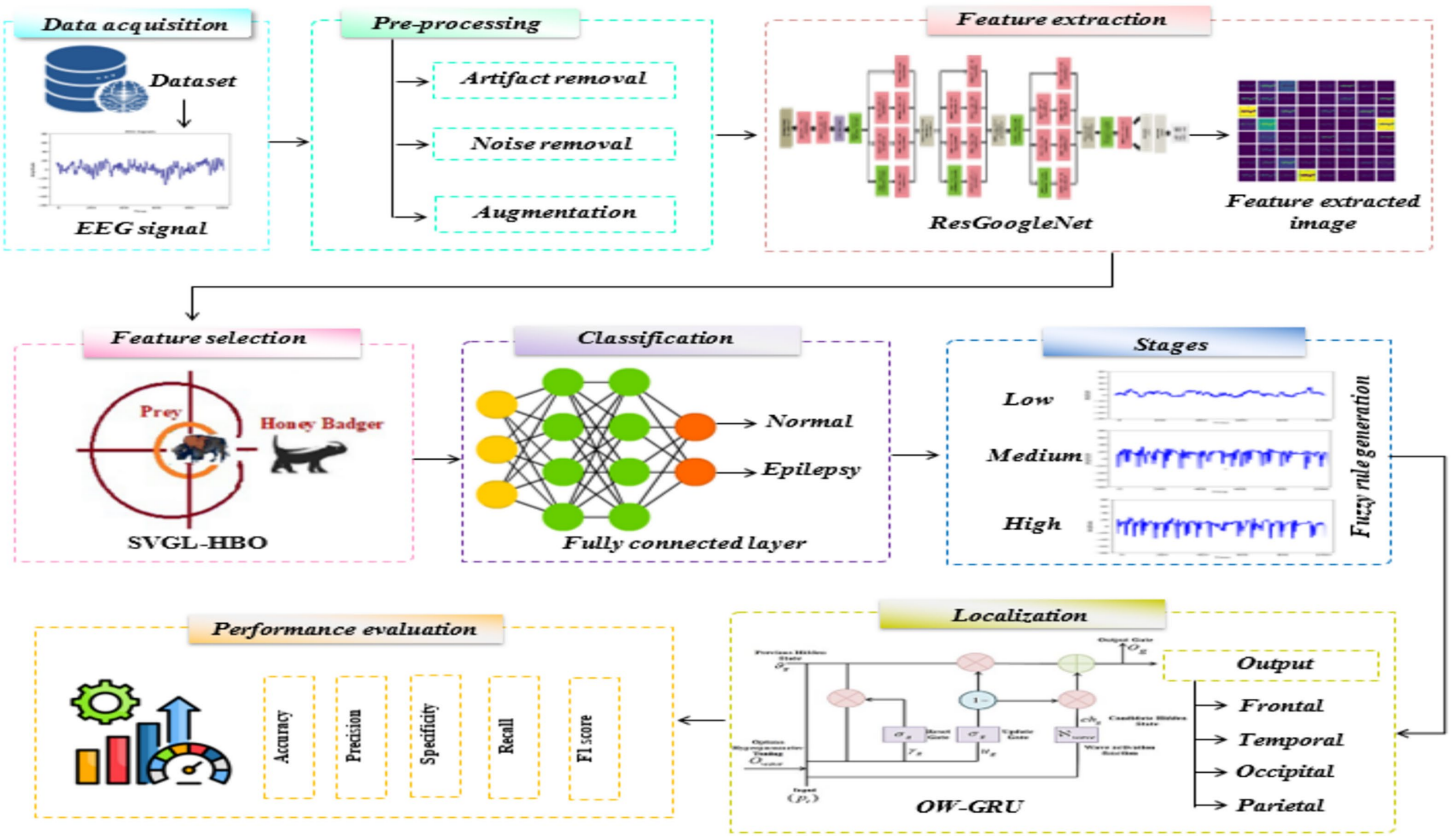
Proposed EPIC-NET methodology.

In this work, a novel deep learning-based EPIC-NET is proposed for epilepsy classification and brain localization using EEG signal. Figure 1 shows the EPIC-NET methodology.

### 3.1 Dataset description

The CHB-MIT scalp EEG dataset was used for experimental evaluation, which included long-term recordings from 23 pediatric individuals with expert-annotated seizure onsets and offsets. A tight patient-wise split was adopted, with 70% of participants assigned to training, 10% to validation, and 20% to testing, and no individuals overlapped. After partitioning, recordings were preprocessed independently using ICA-based artifact removal as well as 30-Hz low-pass filtering, segmented into 2-s windows with 50% overlap, and assigned as seizure or non-seizure using clinical annotations, while inter-ictal segments chosen based on temporal exclusion margins. EEG channels were mapped to frontal, temporal, parietal, and occipital regions to generate lobe-level labels. Data augmentation was only performed to the training set, leaving the validation and test sets unchanged. Hyperparameter tuning of the OW-GRU component was carried out on a separate validation set from the training and test sets. Optuna was used to tune model hyperparameters based purely on the validation-set performance.

### 3.2 Preprocessing

DT-CWT is utilized to denoise EEG signals while preserving critical features required for accurate epilepsy detection and localization. DT-CWT is effective for handling non-stationary EEG signals due to its ability to provide high-quality signal representation and better feature retention. This method employs a multilevel signal decomposition process, making it highly suitable for extracting relevant temporal and frequency-domain features from EEG signals. Importantly, DT-CWT facilitates the detection of wave structures such as epileptic spikes and sharp waves and captures weak periodicities, which are crucial for identifying subtle rhythmic discharges characteristic of epilepsy.

The DT-CWT framework consists of two parallel discrete wavelet transforms (DWTs), the first provides the real and even part of a complex wavelet, while the second offers the real and odd (imaginary) part. These DWTs use separate filter banks, each satisfying the perfect reconstruction (PR) condition, which ensures accurate signal reconstruction and improved analysis for epilepsy-related events. The DT-CWT is mathematically represented by the analytic wavelet function in Equation 1:


(1) $$\Psi (t)=\Psi_{h}(t)+i\;\Psi_{g}(t)$$

Where $\Psi_{h}(t)$ is the real and even component, and $\Psi_{g}(t)$ is the real and odd (imaginary) component of the wavelet. The EEG signals are decomposed into four levels using DT-CWT. At each level, complex detail coefficients are extracted, producing four detail sub-bands D1, D2, D3, D4 as expressed in Equation 2:


(2) $$D1=D1_{\text{real}}+\mathrm{iD}1_{\text{imag}}$$

In a similarly, Equation 3 is used to derive complex approximation coefficients in sub band (A4).


(3) $$A4=A4_{\text{real}}+\mathrm{iA}4_{\text{imag}}$$

In EEG signals, detail coefficients primarily capture high-frequency components that are more susceptible to noise, while approximation coefficients preserve the low-frequency structure of the signal. To suppress noise while preserving essential epileptic features, a thresholding technique is applied to the detail coefficients. This involves using the coefficients D1 to D4 with the original approximation coefficients (A4). The reconstruction process includes up-sampling by a factor of 2 at each decomposition level, followed by the application of synthesis filters, resulting in a clean and feature-rich EEG signal that supports accurate epilepsy detection and localization.

### 3.3 Augmentation

Signal augmentation is used to strengthen the proposed model and reduce class imbalance in training data by boosting signal variability. In this study, EEG signals are subjected to controlled signal-level changes such as noise injection, polarity inversion, time shifting, time stretching, pitch scaling, and random gain. Data augmentation is only applied to the training set after subject-wise data segmentation, while the validation and test sets are left unchanged to ensure an unbiased evaluation. This method improves model generalization while avoiding information leakage and inflating test results. The augmented signals are then translated into time-frequency representations (spectrograms) using the Short-Time Fourier Transform (STFT), which are fed into the proposed EPIC-NET framework. STFT was calculated with a 256-sample Hamming window, an FFT length of 256, and a hop length of 128 (50% overlap), and the study was restricted to the 0–30 Hz frequency range. For multi-channel EEG, spectrograms were created independently for each channel and then averaged to produce a single composite representation. The spectrograms were normalized and scaled to 224 × 224 × 1 before being used in the deep learning model.

### 3.4 Feature extraction

**Figure 2:**
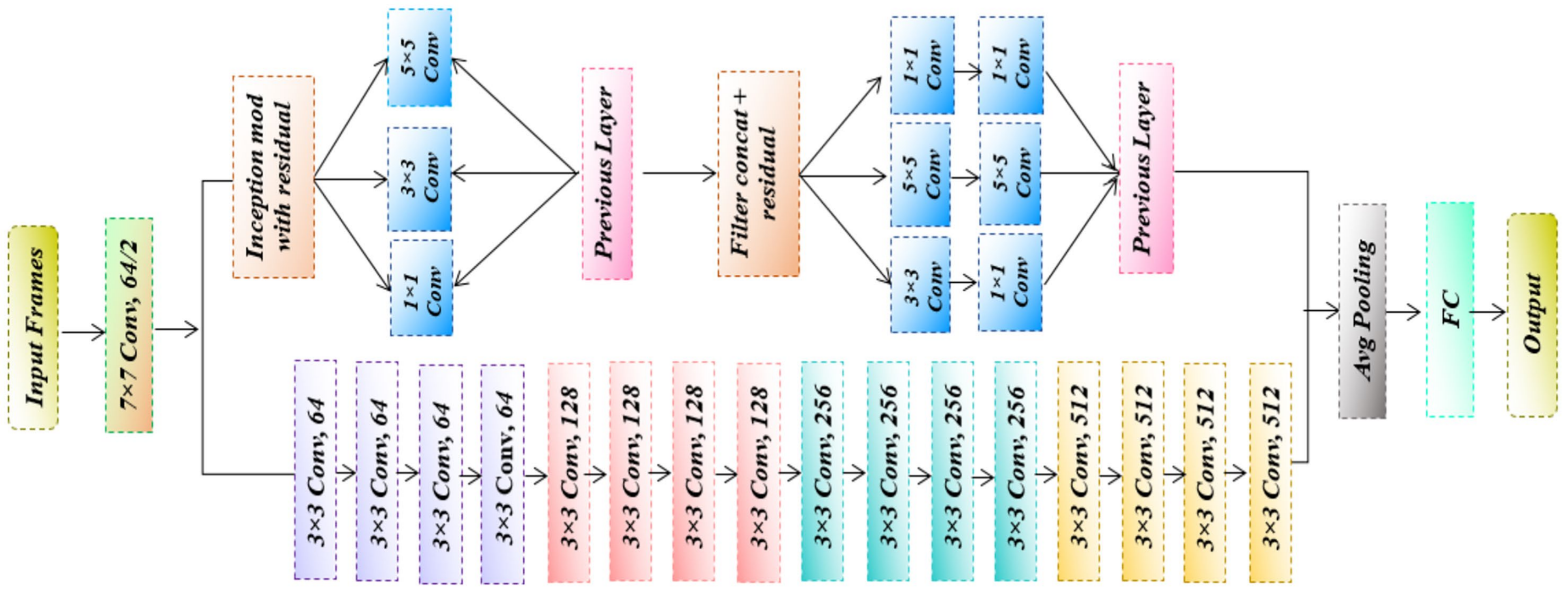
Architecture of ResGoogleNet.

Spectrograms are derived from EEG signals are fed into ResGoogleNet for deep feature extraction, focusing on capturing critical brainwave patterns relevant to epileptic activity detection and localization. The architecture of the modified ResGoogleNet is shown in Figure 2.

The input layer is designed to accept data with dimensions 224 × 224 × 1, corresponding to grayscale spectrogram images. Following the input layer, the convolutional layer values are updated accordingly. In the proposed hybrid model, the last five layers of the original 177-layer architecture were removed and replaced with 10 additional layers, resulting in a modified structure with 182 layers. The model introduces the Inception module concept within a CNN architecture. An Inception module includes operations such as 1 × 1, 3 × 3, and 5 × 5 convolutions, along with 3 × 3 max pooling. The primary purpose of pooling is to reduce the spatial dimensions of the input, thereby lowering computational complexity, although the pooling layer itself has no learning parameters. The 1 × 1 convolution reduce the number of channels, offering additional computational efficiency. A variant of the Inception architecture, known as the Dense-Inception structure, was employed. In this structure, a fully connected dense layer runs parallel to two convolutional layers. While the dense connection ensures complete feature representation, its depth is reduced to limit the number of parameters. The proposed ResGoogleNet architecture is adapted from the original 177-layer GoogleNet model by eliminating the last five pooling and classification layers. Ten new layers are added, including Inception-Residual blocks, a RegNet refinement block, stacked 3 × 3 convolutional layers, and a global average pooling-based classification head, making a total of 182 layers. The Inception-Residual blocks enable multi-scale feature extraction by improving information flow through residual connections, whereas the RegNet block improves seizure-related discriminative features. The stacked convolutional layers and global average pooling refine representations and increase durability for reliable epilepsy detection.

Table 1 summarizes the exact layer-by-layer structure of the updated ResGoogleNet model for EEG-based epilepsy analysis. The design incorporates Inception-Residual blocks as well as a RegNet refinement stage to allow for multi-scale feature extraction and improved discriminative learning. During training, ResGoogleNet performs weight matrix calculation through backpropagation and adam optimizer. These weight matrices, denoted by parameters such as $W_{12}^{n},W_{23}^{n},W_{34}^{n}\;$ are learned representations that transform input features through multiple layers. These matrices store the learned filters of convolutional and dense layers, allowing the network to extract discriminative spatial and temporal patterns from spectrograms for classifying epileptic states. The network iteratively updates these weight matrices to minimize the loss function and improve classification and localization accuracy. Convolutions with various filter sizes (1 × 1, 3 × 3, 5 × 5) are executed in parallel by the Inception module, that allows the network to capture characteristics at various scales. A single method to depict an Inception module is given in Equation 4.


(4) $$\text{Inception}\;(y)=[\begin{array}{l} \mathrm{Con}v_{1\times 1}(y),\mathrm{Con}v_{3\times 3}(y),\\ \mathrm{Con}v_{5\times 5}(y),\text{MaxPoo}l_{3\times 3}(y) \end{array}]$$

**Table 1:** Layer-wise configuration of the proposed ResGoogleNet architecture.

Block name	Kernel size (s)	Number of filters	Activation function
Input layer	–	–	–
Initial convolution	7 × 7	64	ReLU
Inception-residual block 1	1 × 1, 3 × 3, 5 × 5 conv; 3 × 3 max pool	64 (per convolution branch)	ReLU
Inception-residual block 2	1 × 1, 3 × 3, 5 × 5 conv; 3 × 3 max pool	128 (per convolution branch)	ReLU
RegNet refinement block (*n*th stage)	1 × 1, 3 × 3 convolutions with ConvLSTM	Adaptive	ReLU
Convolutional block	3 × 3	64 → 512	ReLU
Global average pooling	–	–	–
Fully connected layer	–	Number of classes	Softmax

where y specifies the input feature map, ${\text{Conv}}_{k\times k}(\cdot )$ indicates a convolution operation with kernel size k×k , and ${\text{MaxPool}}_{3\times 3}(\cdot )$ denotes max pooling. Then along the channel dimension, the outputs are concatenated. Utilizing the Inception module as the residual function ℱ in a residual block is the fundamental principle of ResGoogleNet. The formulation of a ResNet-Inception block is given in Equation 5.


(5) $$X={ℱ}_{\text{Inception}}(y,\{V_{j}\})+y$$

Here, $F_{\text{Inception}}(\cdot )$ represent the Inception module parameterized by weights $V_{j}$ , and X is the output feature map of the residual block. The residual connection adds y straight to the Inception module output, where y is the block’s input. The suggested architecture uses flatten, dense layers, and nine conv2d layers with filter widths of 128 × 64 × 32. For 224 × 224 images, there are 194 × 903 × 073 training samples. The *n*th RegNet function is described by Equations 6–10.


(6) $$Z_{2}^{n}=\mathbb{R}\mathrm{ELU}(Bn(W_{12}^{n}*Z_{1}^{n}+b_{12}^{n})),$$

(7) $$[O^{n},C^{n}]=\mathbb{R}\mathrm{ELU}(Bn(c\mathrm{on}\text{LSTM}(X_{2}^{t},[\;O^{n-1},C^{n-1}]))),$$

(8) $$Z_{3}^{n}=\mathbb{R}\mathrm{ELU}(Bn(W_{23}^{n}*c\mathrm{on\mathbb{C}}\mathrm{AT}[Z_{2}^{n},O^{n}])),$$

(9) $$Z_{4}^{n}=Bn(W_{34}^{n}*Z_{3}^{n}+b_{34}^{n}),$$

(10) $$Z_{1}^{n+1}=\mathbb{R}\mathrm{ELU}(Z_{1}^{n}+Z_{5}^{n})$$

where $Z_{i}^{n}$ indicates the feature representation at the *i*th stage of the *n*th RegNet iteration. $O^{n}$ and $C^{n}$ denotes the hidden and cell states of the ConvLSTM. $W_{\mathrm{ij}}^{n}$ and $b_{\mathrm{ij}}^{n}$ refers to trainable convolutional weights and bias terms, ∗ indicates the convolution operation, and Bn(·) represents batch normalization The correlation distance is denoted by $b_{12}^{n}$ and the 3*3 convolution particles $W_{12}^{n}$ , $W_{23}^{n},$ and $W_{34}^{n}$ have 1*1 kernel. ℬn(.) represents the batch normalization stage, whereas conℂAT(.) denotes the concatenation operation. The enter entity $Z_{2}^{n}$ and the previous output of ConvLSTM $O^{n}$ are the input of conLSTM inside the module. The conLSTM determines whether the data inside the memory cell is given to the $O^{n}$ output hidden characteristic map based on the inputs source. Where, $\tilde{A}$ represents the outcome of the output layer. Some features like peak factor, fuzzy entropy, edges, neuron connectivity are extracted from ResGoogleNet are represented in Equation 11.


(11) $$Y=\{Y_{1},Y_{2},Y_{3},\ldots \ldots \ldots ,Y_{\mathrm{uv}}\}$$

Where, *Y* indicates the selected feature set and $Y_{\mathrm{uv}}$ represents the *u*th feature from the *v*th channel., used for final classification of epileptic states and localization of affected brain regions.

### 3.5 Feature selection

In this phase, SVGL-HBO is used to select the most relevant features from the retrieved features *Y*. The conventional HBA prevents trapping in local optima solutions and offers more reliable solutions by exploring a wider search space. However, it suffers from a slow convergence speed during optimization. So, this research work uses the Stochastic Variance Reduced Gradient Langevin Dynamics in the updation process of the exploration stage. The SVGL-HBO algorithm starts with a population of 30 potential solutions drawn from a uniform random distribution with predetermined lower and upper bounds. The optimization method runs for 100 iterations, with a specified maximum-iteration stopping condition. Each iteration, candidate solutions are updated via stochastic variance-reduced gradient Langevin dynamics. The fitness function is defined using classification performance, and the ultimate fitness is measured in terms of classification accuracy. The optimization is performed on solely training features, and no further convergence-based early stopping is used. Initially, the extracted features *Y* are denoted as population. Then, the position of the population *Q_G_* is derived in Equation 12.


(12) $$Q_{G}=x_{1}*(B_{\mathrm{LOW}}+(B_{\text{HIGH}}-B_{\mathrm{LOW}})),G=1,2,3,\cdots N_{p}$$

Where, *N_p_* represents the number of populations, *B_HIGH_* and *B_LOW_* represent the lower and upper bound of the population, and chosen random number within 0 and 1. Equation 13 describes the fitness function.


(13) $$\chi ={\lambda_{\mathrm{acc}}}^{-}$$

where *λ_acc_^−^* represents the accuracy term. This indicates that the fitness value is directly proportional to the model’s accuracy, meaning higher accuracy results in a higher fitness score.

#### 3.5.1 Exploration stage

The position of the population is updated using Stochastic Variance Reduced Gradient Langevin Dynamics. The estimated stochastic gradient is denoted as ∇*λ* and is defined in Equation 14.


(14) $$Q_{G+1}=Q_{G}-\varpi (\nabla ƛ)+\sqrt{2\varpi }\;\hbar$$

Where, *ϖ* represents the step size, ∇ refers the gradient operator and ℏ represents the standard normal distribution. The variance reduction step is performed as defined in Equation 15,


(15) $$\exists_{\mathrm{vr}}=\nabla ƛ(Q^{\left(Y\right)})-\nabla ƛ(Q^{\left(Y\right)};\varepsilon )$$

Where, $Q^{\left(Y\right)}$ represents the initial position of the honey badger population*. ε* represents the random variable of the Gaussian distribution function. Further, the Langevin Dynamics is updated as in Equation 16,


(16) $$\ell_{\text{update}\;1}=\varepsilon -\varpi \;\exists_{\mathrm{vr}}+\sqrt{2\varpi \;}\hbar$$

Where, *ℓ*_*update*1_ indicates the updated population of the exploration phase.

#### 3.5.2 Exploitation stage

Next, the population position is updated according to the honey phase. During position updating, the new position is accepted if the position has a better value for the fitness value. The updating procedure is expressed in Equations 17–19.


(17) ℓupdate2=∞+μ∗x2∗⊥∗Φ

(18) $$\mu =\{\begin{array}{l} 1\;\;\;,\;\;\;\text{if}\;x_{2}\leq 0.5\\ -1\;,\;\text{else} \end{array}$$

(19) $$⊥=\theta \times \frac{{∠}_{\text{existing}}}{{∠}_{\text{total}}}$$

Where, ∞ represents the best position for the exploitation phase, μ represents the searching solution to find the position, $x_{2}$ represents the randomly chosen value for the second phase within the interval of 0 and 1, ⊥ represents the constant value that adapts with respect to time, θ represents the fixed number, ${∠}_{\text{existing}}$ and ${∠}_{\text{total}}$ represent the existing iterations and the total number of iterations, respectively, and Φ denotes the length separation of the target position. The features selected from the extracted feature set are denoted in Equation 20,


(20) $$p_{t}=\{p_{1},,,p_{2},,,..\ldots ,,,p_{\mathrm{jl}}\}$$

Where, *p_t_* indicates the selected feature set and *p_jl_* denotes the *jl*-number of selected features. Based on the selected features, the FC layer classifies normal and epilepsy classes.

### 3.6 Fuzzy rule generation

The fuzzy algorithm is effective for solving complex, nonlinear problems. However, its performance often depends on carefully designing membership functions and rule sets. To overcome this challenge, a bell-shaped elliptic membership function is adopted which enhances the robustness and simplifies the tuning process of the fuzzy model. In this method, epilepsy activity are classified into Low, Medium, and High based on input features such as alpha (*α*) and delta (*δ*) brain wave signals, and entropy **(℘)** levels. The fuzzy rules for stage classification are defined in Equation 21:


(21) $$\zeta =\{\begin{array}{c} \text{if}\;\alpha \;\text{is high and}\;\delta ,℘\text{is}\;\mathrm{low}\;\;\;\;\mathrm{low}\\ \;\;\text{if}\;\alpha ,\delta ,℘\text{is medium}\;\;\;\text{medium}\\ \text{if}\;\alpha \;\text{is}\;\mathrm{low}\;\text{and}\;\delta ,℘\text{is high}\;\;\mathrm{low}\\ \text{if}\;\alpha ,\delta \;\text{is medium and}℘\text{is}\;\mathrm{low}\;\;\;\text{high} \end{array}$$

Where *α*, *δ*, ℘ represent the alpha waves, delta waves, and entropy features, respectively. After that, the crisp inputs are *μ* changed into fuzzy data with respect to the membership function. The fuzzification process can be calculated as follows, after assigning fuzzy rules, the crisp input features μ are transformed into fuzzy data using a membership function. The fuzzification is calculated in Equation 22:


(22) $$\partial =\frac{\sum \mathbb{R}\times (\zeta )}{ℑ}$$

Where, ∂ represents the fuzzified value, ℝ denotes the rule strength, and ℑ is the membership function of the fuzzy data. The membership function of the bell elliptic is given in Equation 23


(23) $$ℑ=\frac{1}{1+{\left(\frac{|v-\lambda |}{\beta }\right)}^{2\gamma }}$$

Where, *v* represents the membership value in the fuzzy set and *λ* represents the center of the bell curve. *β* represents the width of the elliptical curve. *γ* represents the slope of the elliptical curve. Finally, the defuzzified output representing the epilepsy activity stage (Low/Medium/High) is computed using Equation 24:


(24) $$D_{F}=\frac{\sum \partial \times ℑ}{\sum ℑ}$$

Where, DF represents the defuzzified output for the given fuzzy set. The labeled data is denoted as *Θ*.

Table 2 presents the fuzzy rules used to determine the stages of epilepsy based on alpha, delta, and entropy signal levels. Different combinations of input levels such as low, medium, or high are mapped to corresponding index: Low, Medium, or High. These rules help quantify signal variations in terms of Seizure Activity Index. The fuzzy inference system uses them to infer the stage of brain activity from EEG features. This index is a signal-derived, relative measure that is not proportional to clinically assessed seizure severity.

**Table 2:** Rules for fuzzy inference system.

Rules	Alpha	Delta	Entropy	Stages
1	Low	Low	Low	Low
2	Medium	Medium	Medium	Medium
3	Low	High	High	Low
4	Medium	Medium	Low	High

### 3.7 Classification

**Figure 3:**
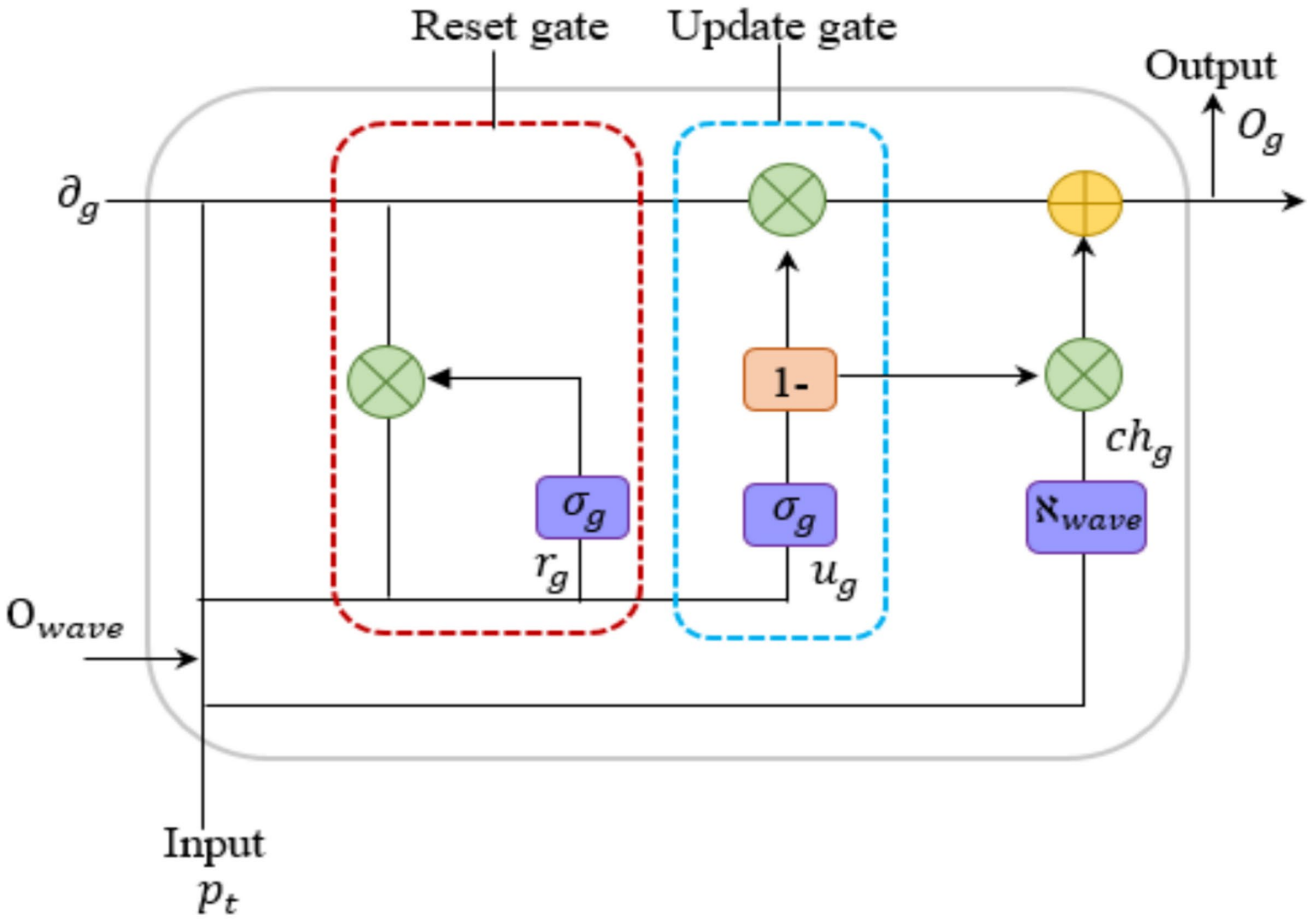
Structure of the OW-GRU approach.

The algorithm of the OW-GRU classifier is outlined in epilepsy classification. The structure of the OW-GRU is displayed in Figure 3.

The conventional GRU model is advantageous for handling complex models as it makes training more efficient and faster. Since GRU is less computational complex, trains more quickly, and has a lower chance of overfitting than LSTM, it was chosen for real-time epilepsy detection. In this study, GRU has been selected over LSTM and Transformer-based models due to its computational efficiency, faster convergence, and suitability for small to medium-sized medical time-series datasets such as EEG. While LSTM is highly effective in capturing long-term dependencies, it introduces additional memory and gate mechanisms that increase training complexity, which can lead to overfitting, especially in limited data scenarios. On the other hand, Transformer-based architectures, though powerful in modeling global dependencies, require significantly more data and computational resources, which are not optimal for real-time clinical EEG processing. GRU offers a simpler structure with fewer parameters while maintaining the ability to capture relevant temporal features. However, GRUs can lead to overfitting issues and they also may experience the vanishing gradient problem during training. To solve these problems, the Optuna hyperparameter tuning technique is included for the overfitting issue. To tackle the vanishing gradient problem, we employ the wave activation function.

However, the use of fixed hyperparameters led to overfitting issues, which were effectively addressed through Optuna-based hyperparameter tuning. The hyper- parameter tuning is given in Equation 25,


(25) $$O_{\text{wave}}=\text{argmi}n_{W\in \tau }(W)$$

$O_{\text{wave}}$ represents the optimal set of tuned hyperparameters, w is the hyperparameter vector and Wϵτ represents the set of hyperparameters to be tuned. From the selected features $p_{t}$ , the reset gate $r_{g}$ calculation is carried out using the sigmoid activation function, which selectively updates memory by determining which information should be discarded or retained. Thus, the $r_{g}$ calculation is given in Equation 26,


(26) $$r_{g}=\sigma_{g}(K_{\mathrm{rg}}.(h_{t-1};p_{t}))$$

Where, $K_{\mathrm{rg}}$ represents the weight in $r_{g}$ obtained from $O_{\text{wave}}$ , $h_{t-1}$ represents the hidden state functions, and $\sigma_{g}$ represents the sigmoidal function. After *r_g_* calculation, the update gate *u_g_* calculation is performed, which regulates how much of the previous memory should be retained and how much of the new input should be incorporated. Hence, the *u_g_* calculation is given in Equation 27,


(27) $$u_{g}=\sigma_{g}(K_{\mathrm{ug}}.(h_{t-1},p_{t}))$$

Where, $K_{\mathrm{ug}}$ represents the weight in ug obtained from $O_{\text{wave}}$ . Then, the candidate hidden state with wave activation function is used instead of the tangent activation to solve the vanishing gradient problem as defined in Equation 28,


(28) $$ch_{g}={ℵ}_{\text{wave}}(K_{ch_{g}}*(r_{g},,h_{t-1},,p_{t}))$$

Where, $K_{ch_{g}}$ represents the weight in $ch_{g}$ obtained from $O_{\text{wave}}$ and ${ℵ}_{\text{wave}}\;$ represents the wave activation function. The wave activation function helps the model in the learning process by allowing the gradients to ow more easily during training, preventing them from getting too small and improving the model’s prediction. The wave activation function is given in Equation 29,


(29) $${ℵ}_{\text{wave}}=\sin(p_{t})$$

Finally, the output gate $O_{g}$ determines which information from the hidden state should be shared to produce the prediction of the model. The output of OW-GRU is expressed in Equation 30,


(30) $$O_{g}=(1-r_{g})*\partial_{g}+r_{g}*ch_{g}$$

At last, the overall classified data is represented as ℜ indicating the localized epileptic activity across brain lobes such as the parietal, temporal, frontal, and occipital lobes. The CHB-MIT dataset does not contain clinically approved lobe-level ground truth labels for seizure localization. As a result, lobe labels are generated using a typical electrode-to-lobe mapping based on EEG channel locations. Seizure segments are classified as frontal, temporal, parietal, or occipital lobes based on dominant activation throughout the relevant electrode groups, with multifocal or generalized cases allocated to the lobe with the greatest average response. As a result, the reported localization results reflect model-inferred regional attribution rather than clinically validated seizure onset localization.

The OW-GRU model has a fixed architecture that includes four GRU layers with 50 units each and a dropout rate of 0.2. Training is done in batches of 128 with a fixed learning rate (0.01 for SGD and the default option for Adam) and weight decay of 1 × 10^−^⁷. Optuna is utilized for limited hyperparameter optimization across 50 trials, with the objective function minimizing the validation mean squared error. Model selection is based on a hold-out validation technique that uses 10% of the training data and does not include k-fold or patient-wise cross-validation.

Table 3 describe the notation and symbol used in the mathematical equation of the EPIC-NET framework. The proposed EPIC-NET framework’s step-by-step workflow for epilepsy detection, activity indexing, and lobe localization are described by Algorithm 1.

**ALGORITHM 1:**
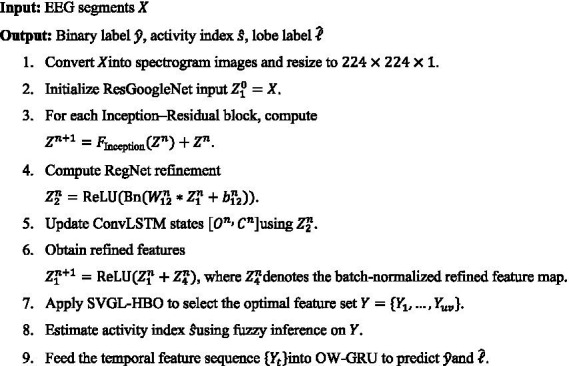
EPIC-NET for epilepsy detection and localization.

**Table 3:** Summary of notation and symbol conventions used in the proposed EPIC-NET framework.

Type	Symbols	Meaning
Scalars	b, *α*, t, *ϵ*, Lval	Bias, learning rate, iteration index, noise term, loss
Vectors	x, z, pt, ht, θ	Input features, layer outputs, GRU inputs, hidden states, solution vector
Matrices/tensors	W, K, Z, O, C	Trainable weights, feature maps, ConvLSTM states
Functions/operators	ReLU(·), BN(·), σ(·), ∇	Activation, normalization, sigmoid, gradient

## 4 Result and discussion

In this section, the experimental setup of EPIC-NET was implemented using MATLAB 2020b and trained using the following experimental setup as shown in Table 1. The EPIC-NET approach was assessed in this section utilizing several parameters including accuracy (AC), specificity (SP), F1 score (F1), precision (PR), MCC and recall (RE) on the gathered EEG Database.

Table 4 outlines the training parameters used for implementing the proposed EPIC-NET model for epilepsy detection and brain localization. The model was developed in MATLAB 2020b on a Windows 10 Pro (64-bit) system with an Intel Core i5 processor and 8 GB RAM. The Children’s Hospital Boston–MIT EEG Database was utilized for training and testing, with a batch size of 140 and a learning rate of 0.0001. Training was carried out for 100 epochs using a composite loss function for both detection and localization. Data augmentation techniques such as noise addition, polarity inversion, pitch scaling, time shifting, time stretching, and random gain were applied to improve model generalization and robustness.

**Table 4:** Training parameters of EPIC-NET.

Parameter	Value
Framework	MATLAB 2020b
Hardware	Windows 10 Pro (64-bit), Intel Core i5, 8 GB RAM
Dataset	Children’s Hospital Boston–MIT EEG Database
Batch size	140
Loss function	Composite loss for detection and localization
Epochs	100
Learning rate	0.0001
Augmentation	Noise addition, polarity inversion, pitch scaling, time shifting, time stretching, random gain

**Figure 4:**
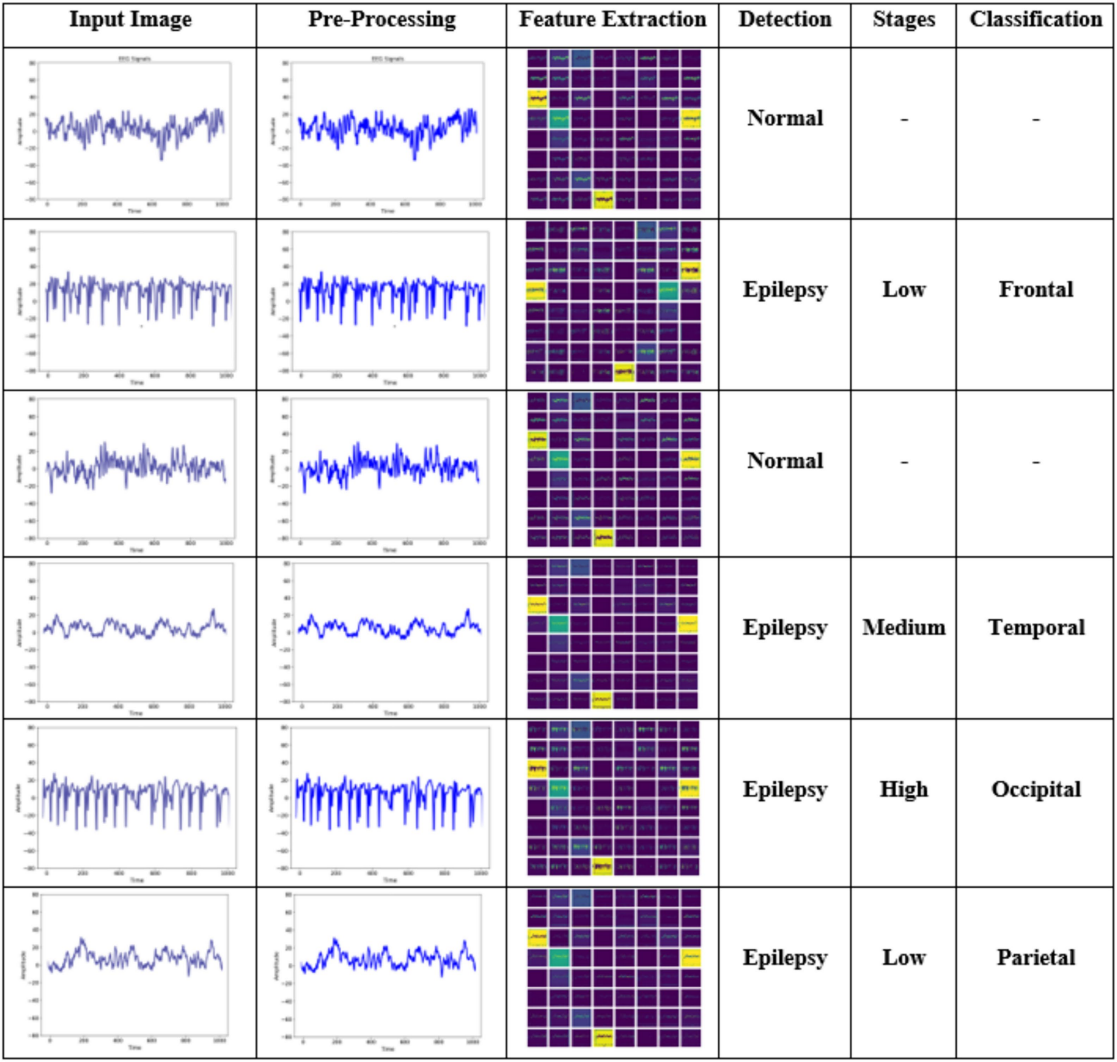
Experimental result of the proposed EPIC-NET.

Figure 4 shows the simulation results of the proposed EPIC-NET using different input samples. Column 1 presents the EEG signal and Column 2 shows the pre-processed images. Column 3 displays the extracted feature maps highlighting key frequency and entropy patterns used for accurate stage identification. Column 4 shows the detection result, distinguishing between normal and epileptic conditions. Column 5 indicates the epilepsy stage like Low, Medium and High based on the features. Column 6 presents the localized brain region affected, classified into Frontal, Temporal, Occipital, and Parietal regions depending on the stages.

### 4.1 Performance analysis

The performance of the EPIC-NET is evaluated over the standard parameters namely specificity (SP), accuracy (AC), precision (PR), recall (RE), F1-score (F1), and Matthews correlation coefficient (MCC), as defined in Equations 31–36.


(31) $$\mathrm{SP}=\frac{\mathrm{TRN}}{\mathrm{TRN}+\mathrm{FAP}}$$

(32) $$\mathrm{AC}=\frac{\mathrm{TRP}+\mathrm{TRN}}{\mathrm{TRP}+\mathrm{TRN}+\mathrm{FAP}+\mathrm{FAN}}$$

(33) $$\mathrm{PR}=\frac{\mathrm{TRP}}{\mathrm{TRP}+\mathrm{FAP}}$$

(34) $$\mathrm{RE}=\frac{\mathrm{TRP}}{\mathrm{TRP}+\mathrm{FAN}}$$

(35) $$F1=2(\frac{\mathrm{PR}*\mathrm{RE}}{\mathrm{PR}+\mathrm{RE}})$$

(36) $$\mathrm{MCC}=\frac{\mathrm{TRP}\times \mathrm{TRN}-\mathrm{FAP}\times \mathrm{FAN}}{\sqrt{\begin{array}{l} \left(\mathrm{TRP}+\mathrm{FAP})\times (\mathrm{TRP}+\mathrm{FAN}\right)\\ \times (\mathrm{TRN}+\mathrm{FAP})\times (\mathrm{TRN}+\mathrm{FAN}) \end{array}}}$$

Where TRP, TRN, FAP, and FAN denote true positive, true negative, false positive, and false negative, respectively.

Table 5 displays the detection performance attained by the proposed EPIC-NET for Epilepsy classification and Brain Localization. PR, F1, SP, AC, MCC, and RE are metrics that determine the overall performance of EPIC-NET. The proposed EPIC-NET achieves a total accuracy of 98.80% on the EEG database. The EPIC-NET also achieves 96.23, 96.41, 96.94, 97.43, and 97.11% overall PR, F1, SP, MCC and RE.

**Table 5:** Performance assessment of the EPIC-NET.

Types	AC	PR	RE	SP	F1	MCC
Normal	99.07	96.56	97.45	97.00	96.50	97.50
Epilepsy	98.54	95.91	96.77	96.88	96.32	97.36
Overall	98.80	96.23	97.11	96.94	96.41	97.43

**Figure 5:**
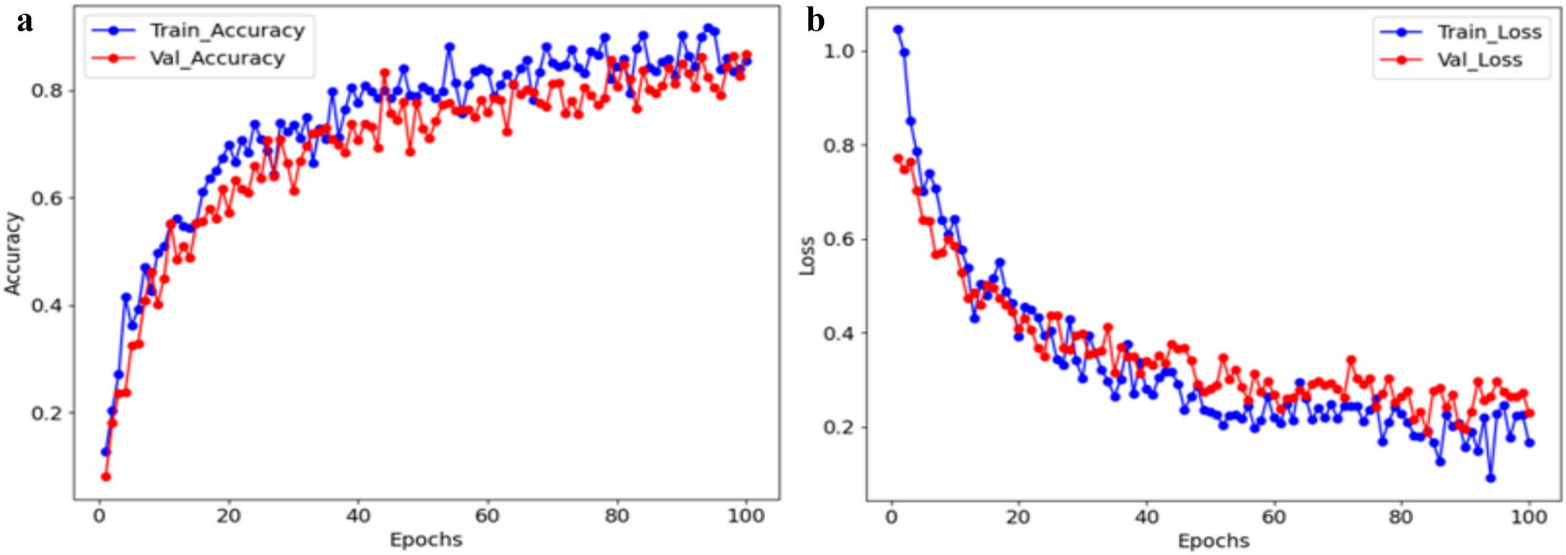
**(a)** Accuracy and **(b)** loss graph of the EPIC-NET.

Figure 5a shows an accuracy curve by epochs on the x-axis and AC rate on the *y*-axis. A high accuracy level of 98.80% based on the epochs is shown by the training and testing AC curves. Figure 5b displays the loss curve, with epochs on the x-axis and the loss rate on the *y*-axis. During training, the loss of the model decreases over time, reflecting improvement in learning. Since, the EPIC-NET operates well during the testing and training stages.

**Figure 6:**
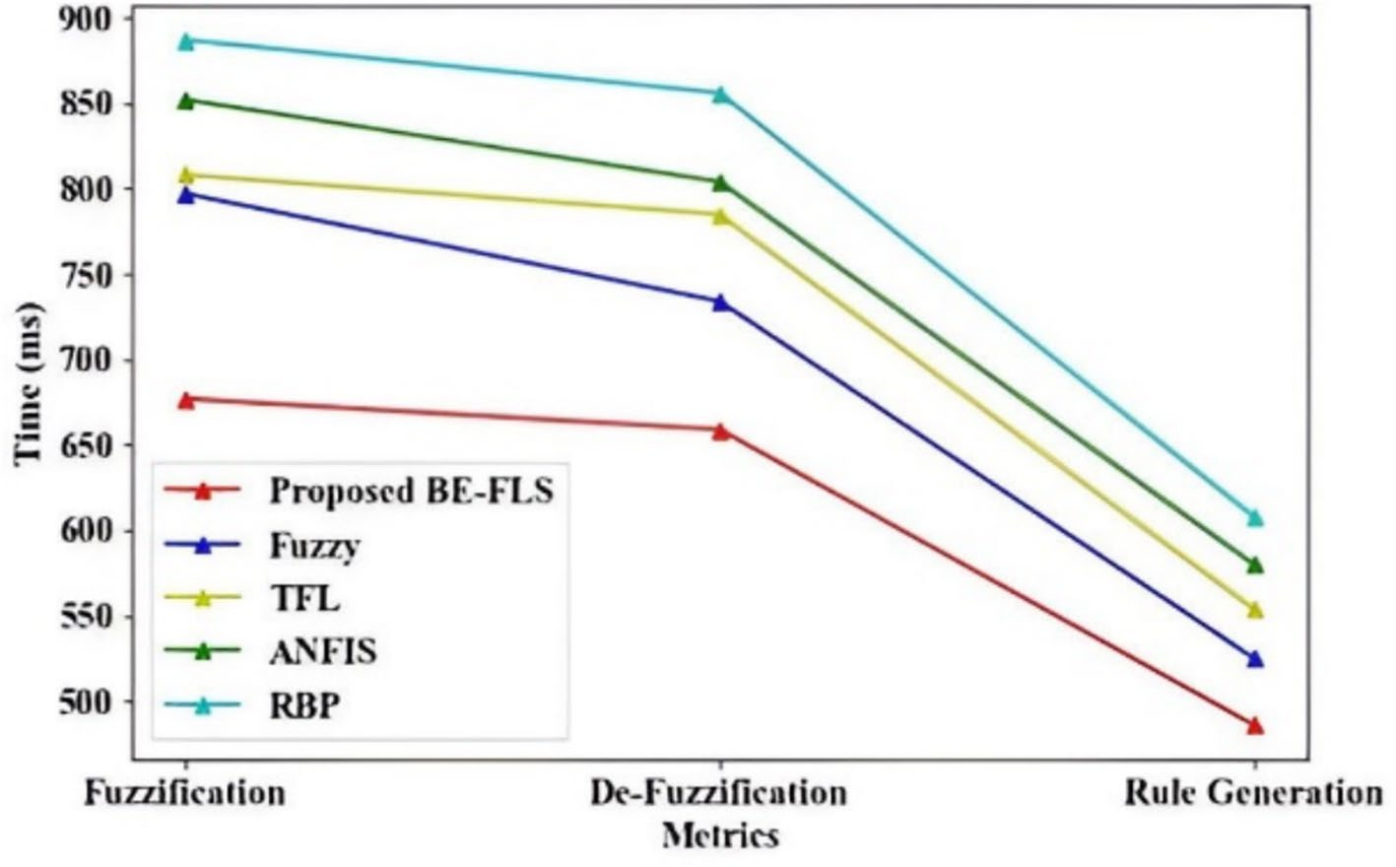
Performance analysis of the proposed BE-FLS regarding fuzzification, defuzzification, rule generation time.

**Figure 7:**
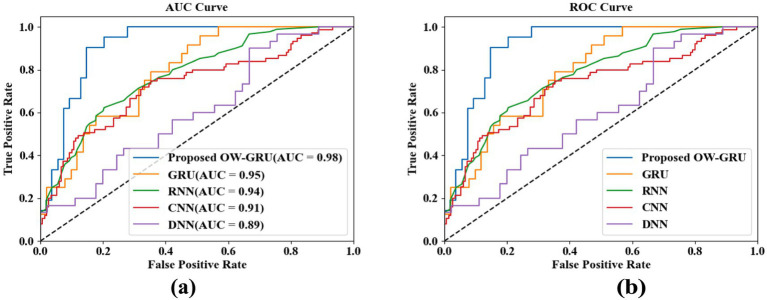
Evaluation of model performance. **(a)** AUC curve and **(b)** ROC curve.

Figure 6 displays the performance analysis of the suggested BE- FLS regarding fuzzification, de-fuzzification, and rule generation time. In Figure 7, the proposed BE-FLS achieves a fuzzification, de-fuzzification, and rule generation time of 677 ms, 659 ms, and 486 ms, respectively. However, the existing methods obtained an average fuzzification, defuzzification, and rule generation time of 808 ms, 785 ms, and 554 ms, respectively. The tuning problem in the membership function and rules of the fuzzy algorithm is solved by using the bell elliptic membership function in the proposed approach. Therefore, the proposed methodology has low time complexity when compared to the current models.

The proposed OW-GRU model performs better compared to baseline models such as DNN, CNN, RNN, and GRU under the adopted experimental protocol for detecting and localizing epileptic activity as shown in Figure 7. Figure 7a reports a high AUC of 0.98, indicating strong class separation and Figure 7b shows the ROC curve, where OW-GRU demonstrates higher sensitivity and specificity across thresholds.

**Figure 8:**
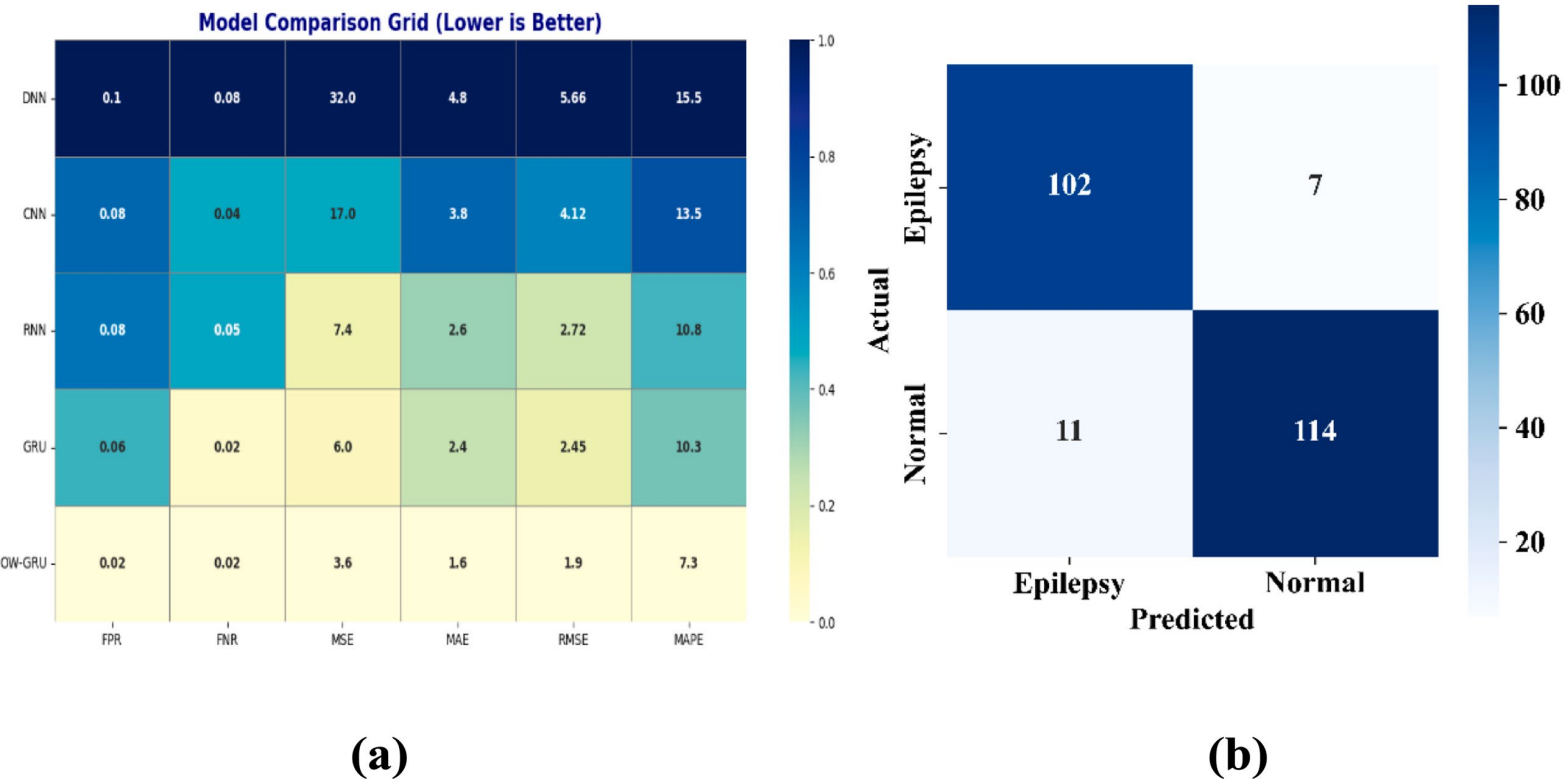
Confusion matrix **(a)** location identification performance **(b)** binary classification.

Figure 8a shows the OW-GRU technique demonstrates improved performance than the existing methods like GRU, RNN, CNN, DNN with lower FPR of 0.02381 and FNR of 0.015625 values. It also achieves lower error rates: MSE of 3.6, MAE of 1.6, RMSE of 1.89, and MAPE of 7.3. Optuna-based hyperparameter tuning reduces overfitting, enabling better algorithmic differentiation of epileptic patterns across brain-lobe locations. Figure 8b shows the confusion matrix for the binary epilepsy classification test. The model successfully classifies the majority of epileptic and normal EEG segments, with few misclassifications in either class. These findings indicate that the proposed technique performs reasonably well for seizure detection on the examined test data.

### 4.2 Ablation studies

To investigate the role of various modules in the proposed EPIC-NET framework, ablation research was carried out by methodically deleting one component at a time from the entire pipeline. The entire configuration includes EEG denoising, spectrogram-based time-frequency representation, deep CNN (ResGoogleNet) feature extraction, SVGL-HBO feature selection, and a fuzzy activity indexing step followed by temporal modeling. To guarantee a fair comparison, the remaining preprocessing processes, network architecture, training method, and hyperparameters were maintained same for each ablation setting. Table 6 shows the ablation analysis of the EPIC-NET pipeline. Dropping DT-CWT denoising, SVGL-HBO feature selection, or the fuzzy activity index consistently reduces performance, which indicates that each component positively contributes to overall detection accuracy.

**Table 6:** Ablation study evaluating the contribution of key components in the proposed EPIC-NET framework.

Configuration	ACC (%)	F1 (%)	MCC (%)
Full EPIC-NET (proposed)	98.7	98.4	97.9
Without DT-CWT denoising	97.1	96.7	96.0
Without SVGL-HBO feature selection	95.6	95.2	94.3
Without fuzzy activity index	96.4	96.0	95.1

### 4.3 Comparative analysis

The proposed EPIC-NET was determined to be the most efficient method after evaluating the effectiveness of previous methods. The efficiency was calculated using the specific metrics for detect the presence of epilepsy.

According to Table 7, the proposed ResGoogleNet is higher than that of the classic networks like DenseNet, AlexNet, GoogleNet, and ResNet. The proposed ResGoogleNet improves its accuracy by 1.46, 4.17, 2.67, and 0.66% better than DenseNet, AlexNet, GoogleNet, and ResNet, respectively.

**Table 7:** Comparison evaluation of different DL network.

Techniques	AC	F1	PR	RE	SP
DenseNet	97.37	90.84	94.21	86.26	88.53
AlexNet	94.84	94.59	91.03	93.37	92.38
GoogleNet	96.23	93.62	89.61	80.83	90.42
ResNet	98.15	95.07	93.49	95.18	94.74
Proposed ResGoogleNet	98.80	96.41	96.23	97.11	96.94

**Figure 9:**
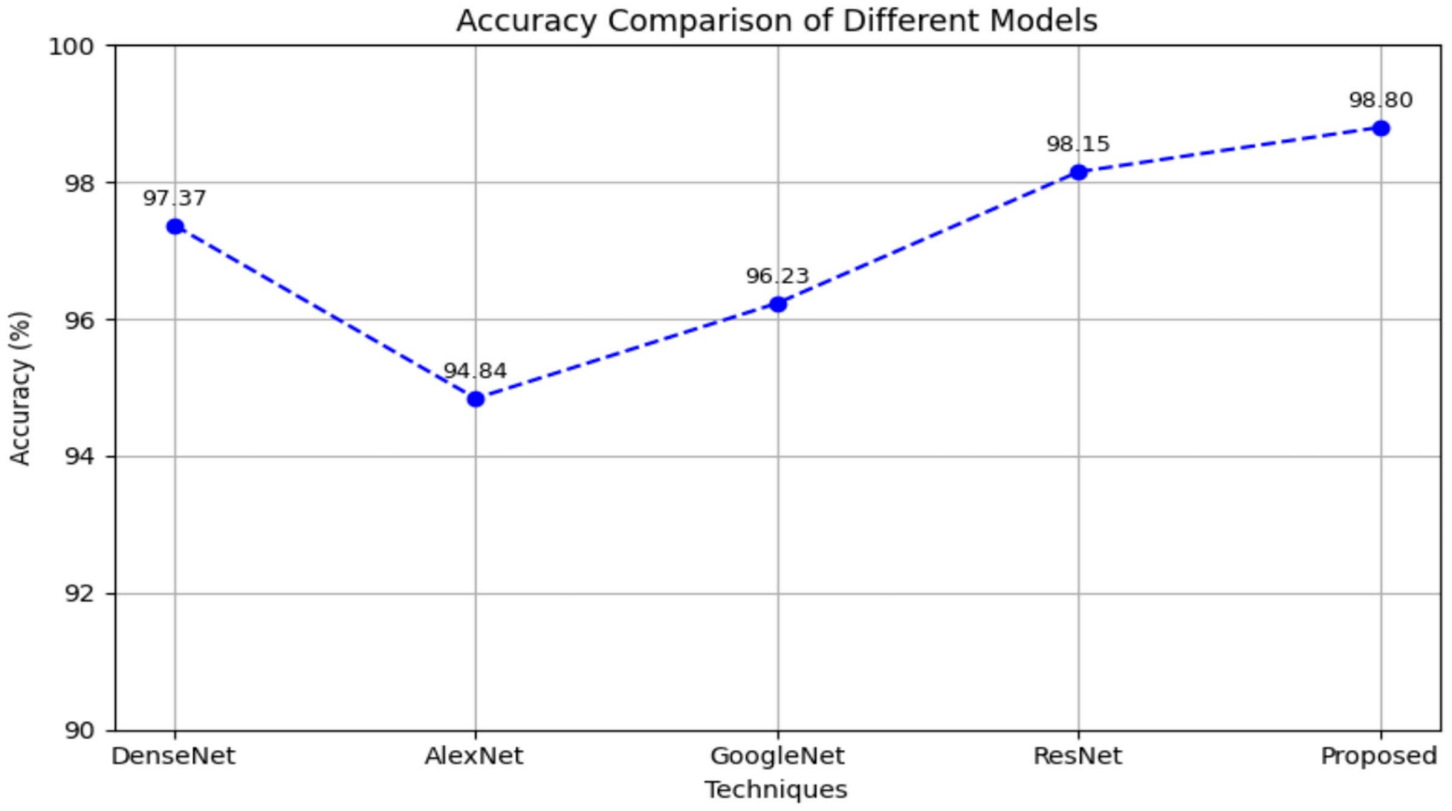
Accuracy comparison of different models.

Figure 9 illustrates the performance of various DL models used for epileptic seizure detection. Among the DenseNet, AlexNet, GoogleNet, and ResNet, the proposed model attains the highest AC of 98.80%, indicating that the EPIC-NET approach effectively capturing relevant EEG features for seizure detection.

Table 8 shows the performance of the proposed with various existing classifiers in terms of AC, TPR, and TNR, and the values are expressed in mean and standard deviation. Comparing the proposed OW-GRU methodology to existing approaches, the mean and standard accuracy value are higher at 98.8 ± 0.89. The accuracy of the proposed OW-GRU strategy is 98.8%, greater than that of the traditional methods.

**Table 8:** Performance comparison of the proposed with various existing models.

Technique	AC	TPR	TNR
DNN	98.1 ± 0.009	98.4 ± 0.007	97.6 ± 0.132
CNN	96.2 ± 2.94	98.1 ± 0.07	94.1 ± 3.85
RNN	94.5 ± 3.83	94.3 ± 3.97	92.5 ± 5.35
GRU	91.8 ± 5.69	92.6 ± 5.01	84.6 ± 11.65
Proposed OW-GRU	98.8 ± 0.89	98.7 ± 0.91	98.1 ± 1.37

**Figure 10:**
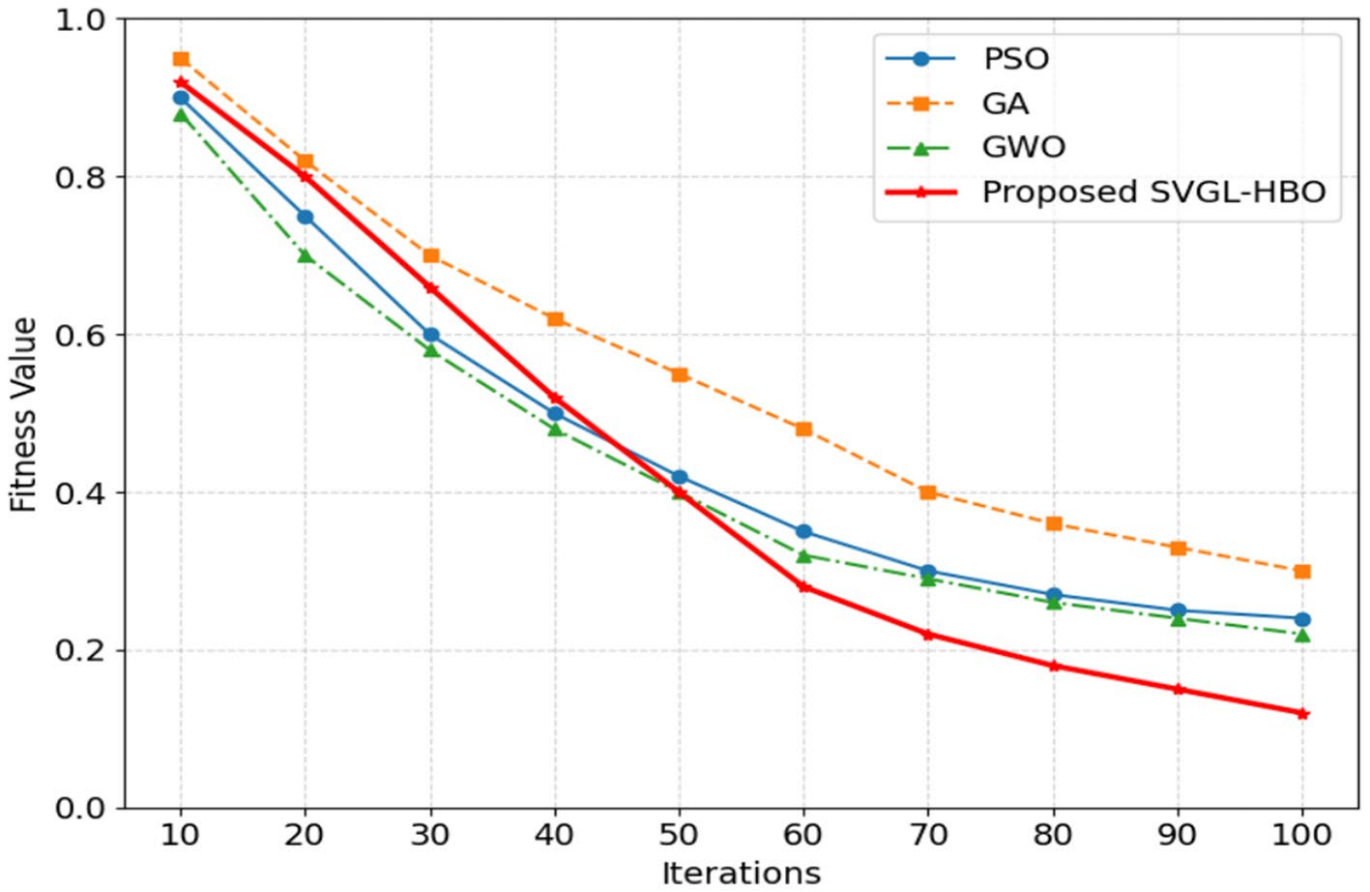
Convergence graph of the proposed SVGL-HBO algorithm.

Figure 10 demonstrates the convergence comparison of the proposed algorithm vs. existing algorithms. The proposed SVGL-HBO algorithm compared with existing algorithms like Genetic Algorithm (GA), Particle Swarm Optimization (PSO) and Grey Wolf Optimizer (GWO) for feature selection. The results demonstrate that SVGL-HBO achieves faster convergence and identifies optimal feature subsets more effectively. The proposed SVGL-HBO algorithm starts with a population of 30 potential solutions drawn from a uniform random distribution with predetermined lower and upper bounds. The optimization method runs for 100 iterations, with a specified maximum-iteration stopping condition. Each iteration, candidate solutions are updated via stochastic variance-reduced gradient Langevin dynamics. The fitness function is defined using classification performance, and the ultimate fitness is measured in terms of classification accuracy. The optimization is performed on solely training features, and no further convergence-based early stopping is used.

Table 9 compares the proposed EPIC-NET framework to selected EEG-based seizure detection research using the CHB-MIT dataset to place it in context. Direct numerical comparison is limited to studies that use explicit patient-independent (patient-wise) evaluation methodologies. Studies with different or not well-defined experimental settings are thus discussed qualitatively rather than using formal numerical comparison. The EPIC-NET approach provides good classification accuracy for epilepsy detection and lobe-level pattern distinction on the CHB-MIT dataset. The EPIC-NET model improves the overall accuracy by 5.92, 10.02, and 0.59% compared to RNN, SVM and CNN, respectively. The proposed EPIC-NET model demonstrates the comparison of the accuracy is better than the existing models.

**Table 9:** Accuracy comparison of existing approaches and proposed approach.

Study	Method	Evaluation protocol	Test augmentation	Evaluation level	Reported performance
Liu et al. (2022)	CNN-BiLSTM with channel perturbation	Patient-independent	Not stated	Segment-level	Accuracy: 97.51%, AUC: 90.82%
Aboyeji et al. (2025)	Time-frequency DL (LOPOCV)	Leave-one-patient-out	No	Segment-level	Accuracy: 87.29 ± 10.48%
Ali et al. (2024)	Cross-subject DL model	Subject-wise	No	Segment-level	Sensitivity: ~75%
Proposed EPIC-NET	MTL + Neuro-symbolic	Strict patient-independent	No	Segment-level	Accuracy: 98.80%, MCC: 97.43%

## 5 Conclusion

This research proposed a novel EPIC-NET for epilepsy detection and localization using EEG signal dataset. The DT-CWT is employed for denoising the EEG signals. Signal augmentation techniques such as, time stretching, pitch shifting and noise injection are applied to EEG signals to balance class distribution and enhance the model generalization across different epileptic patterns. The augmented spectrograms are fed into ResGoogleNet for deep feature extraction, emphasizing critical brainwave patterns relevant to detecting epileptic activity and identifying affected brain regions. SVGL-HBO algorithm is utilized for feature selection and FC layer is used to detect the presence of epilepsy. A BE-FLS is utilized to produce an interpretable Seizure Activity Index divided into Low, Medium, and High levels based on the EEG-derived features. The OW-GRU integrates GRU with wavelet-based preprocessing to capture both temporal and frequency-domain features from EEG signals. Optuna optimizes hyperparameters automatically, enhancing the GRU’s performance and reducing overfitting for epilepsy localization in brain lobes. The proposed EPIC-NET model also achieves 96.23, 96.41, 96.94, 97.43, and 97.11% overall PR, F1, SP, MCC, and RE. The EPIC-NET increases the overall AC by 5.92, 10.02, and 0.59% better than RNN, SVM and CNN, respectively. While the proposed methodology demonstrates potential findings, the Seizure Activity Index and lobe-level localization are generated from computational EEG patterns and require further clinical validation before application in diagnostic or surgical decision-making. In the future, the EPIC-NET can be evaluated on larger and more diverse EEG database to improve its robustness. Additionally, integration with edge computing platforms could enable real-time, on-device epilepsy detection and localization.

## Data Availability

The original contributions presented in the study are included in the article/supplementary material, further inquiries can be directed to the corresponding author.
